# Perception is not all-purpose

**DOI:** 10.1007/s11229-018-01937-5

**Published:** 2018-09-17

**Authors:** Bence Nanay

**Affiliations:** 1 University of Antwerp, D 413 Grote Kauwenberg 18, 2000 Antwerp, Belgium; 2 Cambridge University, Cambridge, UK

**Keywords:** Perception, Attention, Intention, Cognitive penetration

## Abstract

I aim to show that perception depends counterfactually on the action we want to perform. Perception is not all-purpose: what we want to do does influence what we see. After clarifying how this claim is different from the one at stake in the cognitive penetrability debate and what counterfactual dependence means in my claim, I will give a two-step argument: (a) one’s perceptual attention depends counterfactually on one’s intention to perform an action (everything else being equal) and (b) one’s perceptual processing depends counterfactually on one’s perceptual attention (everything else being equal). If we put these claims together, what we get is that one’s perceptual processing depends counterfactually on one’s intention to perform an action (everything else being equal).

## Beyond the cognitive penetrability debate

One of the fashionable debates in philosophy of perception these days is about the cognitive penetrability of perception. The question is whether beliefs and knowledge influence perception. There has been much discussion about what any of these terms mean: does ‘perception’ here mean perceptual experience or perceptual processing? If the latter, what level of perceptual processing? Does the ‘cognitive’ here have to mean high-level belief or knowledge? Can it be an emotional state (Schupp et al. 2004; Schmitz et al. 2009; Pessoa and Ungerleider 2005)? A desire (Stokes 2012)? A state further up in the hierarchy of perceptual processing, regardless of whether it merits the label ‘belief’ (see Teufel and Nanay 2017 for summary)?

The aim of this paper is to move past the cognitive penetrability debate and explore how perception depends on the action we want to perform, regardless of whether it depends on beliefs and knowledge. My claim is that even if we deny that perception is cognitively penetrated, we still have strong reason to doubt that perception would be all-purpose.

Suppose that I look at my phone, I form a belief that I have a phone in front of me, I have a desire to call a cab, I form an intention to call a cab and then I call a cab:

- Perceiving X → Belief (X) → Desire to Q → Intention to Q → Q-ing.

The cognitive penetrability debate is about the interaction between the first two mental states: about how perceiving x and the belief it gives rise to interact and whether the latter influences the former.

- Perceiving X ← → Belief (X) → Desire to Q → Intention to Q → Q-ing.

What I’m interested in is whether the mental states further down the line influence perception. And even if all claims about the cognitive penetrability of perception turn out to be false, we still have plenty of evidence that the action we want to perform influences perception (see Sect. 3). So if I’m looking for a phone because I’m trying to call a cab, the ‘Perceiving X’ will be very different from what it would be if I were looking for something to drive a nail into the wall with.
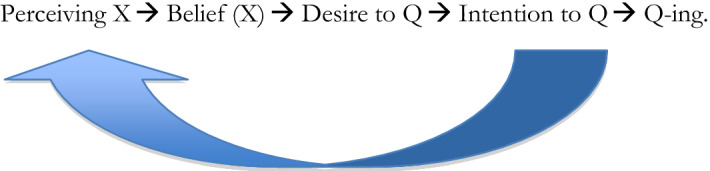


My claim is that perceptual processing depends counterfactually on the action we want to perform. In other words, perceptual processing varies as the action one wants to perform varies. Most philosophical and psychological theories of perception, regardless of which side they are on in the cognitive penetrability debate, claim or at least assume the opposite. Let’s start with some by now somewhat archaic accounts of the human mind.

Modularist theories of perception claim that perception is an encapsulated process that is impenetrable for higher order cognitive processes (see, for example, Fodor 1983; Marr 1982; Pylyshyn 1999). The general idea is that the perceptual system is an ensemble of encapsulated modules and these modules send their outputs to the central processing unit, which then pulls them together, does some calculations and then sends some kind of output. According to this picture, one’s intention to act would not have any influence on one’s perception as perception is encapsulated not only from the central processing unit (from beliefs and knowledge) but also from anything the output unit does.

Interactionist theories of perception, on the other hand, insist that perception is not impenetrable; higher order cognitive processes do influence our perception. These theories say very little about the interaction between beliefs and intentions. While they allow for beliefs influencing perception, they do not talk about the possibility of intentions influencing beliefs (and thereby influencing perceptions) (Gregory 1966; Rock 1983; see also Hommel et al. 2001 for a detailed discussion of this.).

A somewhat odd and rightly frowned upon variety of the interactionist theories of perception is J. J. Gibson’s theory of direct perception. But Gibson also claims explicitly that what we see does not depend on our intention to perform an action. According to him, I perceive a post box as affording posting a letter each time I perceive it, regardless of whether I have a letter to post in my pocket (Gibson 1966, p. 228, p. 246, 1979, pp. 138–139).

In general, the received view is that perception is all-purpose: what we intend to do does not influence what we see.^1^ The perceptual system churns out its output regardless of what the action system does. And the action system has to rely on the action-neutral all-purpose representations that the perceptual system provides. This is the ‘all-purpose perception view’.

I will to argue that this ‘all-purpose perception view’ is wrong (see also Nanay 2006). In other words, perceptual processing does sometimes vary as the action one intends to perform varies. To put it very simply, what we see does sometimes depend on what we want to do. Perception is not all-purpose.

## The structure of my proposal

Before we begin, I need to clarify what I mean by the claim that perceptual processing depends counterfactually on the action one intends to perform.

First of all, what does ‘intention’ mean? One major move in the 1980s in the philosophy of action literature was to distinguish two different kinds of intentions, proximal versus distal (Mele 1992), present-directed versus future-directed (Bratman 1987), immediate versus prospective (Brand 1984), prior versus intention-in-action (Searle 1983). These distinctions are all somewhat different,^2^ but they all agree that proximal intentions (or immediate intentions or present-directed intentions or intentions-in-actions), to quote Mele, ‘trigger appropriate actional mechanisms’ (Mele 1992, p. 177, see also Mele 2003, p. 55).^3^

So the very general picture of the initiation of intentional actions is the following. Distal intentions (together with some representational states about the action-relevant features of the environment) cause proximal intentions. And proximal intentions then cause the action itself. There are many ways in which this picture needs fine-tuning, and much of the last decades of philosophy of action was taken up with that task. What is the content of distal intentions? Does it need to be the same as the content of proximal intentions? And does the intentional action of Q-ing need to be caused by the distal intention with the content ‘to Q’?

This picture has some attractive features. For example, it can give at least a partial answer to the problem of deviant causal chains. There are some examples of actions where the agent does have a distal intention to Q and she does perform Q, but because the causal link between the distal intention and the action is somewhat unusual, the action is not intentional. One widely repeated example is of the nervous robber, who is supposed to signal the start of the robbery at a part by spilling his drink, but when the moment comes, this makes him so nervous that his hand starts shaking and he spills his drink as a result (Frankfurt 1978, p. 157). We have a distal intention and a successfully performed action, but the action is, nonetheless, not intentional (and maybe not even an action) because the causal chain between the two is deviant. One way (definitely not the only way) of zooming in on what is wrong with the causal chain in this example (and the vast quantities of examples of a similar kind proposed in the literature, see, e.g., Brand 1984, pp. 17–18; Davis 1984, p. 113; Mele 1987, p. 56, 1992, p. 182; Davidson 1980, p. 78) is to assert that in order for an action to be intentional, it is not enough if it is caused by a distal intention to perform it (or to perform some action-type it is a determinate of), it needs to be caused by an appropriate proximal intention, which, in turn, was caused by an appropriate distal intention. In other words, the causal chain is deviant because it does not go through the appropriate proximal intention.

I will use the term ‘intention to Q’ in this to refer to what Mele calls proximal intention to Q. So we should re-draw the figure from the previous section accordingly:
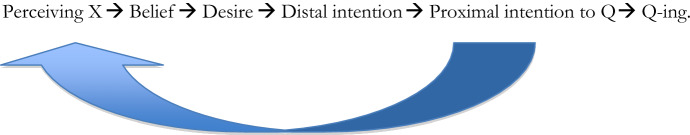


More clarifications: what is perceptual processing? It is important that this paper is about how intentions influence perceptual processing and not about how they influence perceptual experience. I have no idea how we can do rigorous philosophizing (or rigorous empirical research) about perceptual experiences as I see no good criteria for keeping apart perceptual and non-perceptual experiences in a way that does not rely on notoriously unreliable introspective reports (see Nanay 2012a, b, 2013).

What is perceptual processing then? It is processing in the perceptual system. What is the perceptual system? And how do we keep it apart from post-perceptual processing? This question is less hopeless than the one about perceptual phenomenology. Some parts of the processing of the sensory input are very clearly perceptual. Early cortical processing, for example, processing in the primary and secondary visual cortices and in V4/V8 or MT would count as perceptual processing. If we go further up, the answer is not so clear (see Teufel and Nanay 2017; Nanay 2017, 2018 for more detailed treatments of the question about where perceptual processing ends and post-perceptual processing begins). But I will argue that even if we restrict perceptual processing to early cortical processing, we can still conclude that perceptual processing depends on our proximal intentions.

Finally, what does the ‘counterfactual dependence’ of one’s perceptual processing on one’s intention to perform an action mean? The advocates of the ‘all-purpose view’ would, of course, agree that the intention to perform an action at t_1_ does influence my perceptual processing at t_2_, if I do indeed perform this action and if t_2_ follows t_1_. For example, my intention to turn my head at t_1_ (if executed) obviously does influence my perceptual processing in the next moment. There are some other fairly obvious examples of action-perception dependence that the ‘all-purpose view’ would accept. When, for example, one is perceiving one’s own action, perceptual processing clearly depends on the action one performs, thus presumably it also depends on one’s intention to perform this action.

To rule out these irrelevant cases, we should say that according to the ‘all-purpose view’, perceptual processing does not depend on what action one intends to perform, *everything else being equal*, that is, importantly, *even if one’s sensory stimulation is still the same* (where I take sensory stimulation to be a physiological state: a retinal image, for example).

In all the examples I gave in the last paragraphs, the action the agent intends to perform influences her sensory stimulation, thus, her perceptual processing. For example, when I am looking at my hand while reaching out to take a sip from a glass and when I am looking at my hand while ringing the doorbell, my sensory stimulation will be different in the two cases, therefore, it is not surprising that perceptual processing will also be different. The intention to perform a certain action influences the sensory stimulation and the sensory stimulation influences perceptual processing. The ‘all-purpose view’ would not deny this.

What the ‘all-purpose view’ would deny is that it is possible that perceptual processing varies with the action one intends to perform even if the sensory stimulation is the same. Their claim is that when I am looking at a glass of wine while intending to drink it, I will have the same perceptual processing as I would if I were looking at the same glass of wine while intending to pour it out under the table (supposing that my sensory stimulation is the same in the two situations).

I aim to argue against this view. I will argue that perceptual processing depends counterfactually on the action one intends to perform even if the sensory stimulation is the same. In other words, if the action I intend to perform were different, perceptual processing would be different, even if my sensory stimulation were the same.

## Empirical considerations

Denying the all-purpose view is not such a radical claim. Some held similar views. William James famously said that “attention […] out of all the sensations yielded, picks out certain ones as worthy of notice and suppresses all the rest. We notice only those sensations which are signs to us of *things* which happen practically […] to interest us”. (James 1892/1961, p. 39).

More recently, there are empirical findings that strongly suggest that the intention to perform an action influences very early stages of perceptual processing. First I want to set aside a couple of widely publicized results that one may be tempted to use here, but really shouldn’t.

The first one is a highly popularized experiment by Henk Aarts, Ap Dijksterhuis and Peter De Vries (Aarts et al. 2001), which is intended to show that being thirsty (which may sound like it has a lot to do with having a proximal intention (to drink)) makes one more ‘perceptually ready’ (which may sound like it has a lot to do with perceptual processing). Even if we ignore the replication crisis that hit the lab where this result was found especially hard, this claim is deeply problematic.

The main problem is that the tasks where thirsty and non-thirsty individual showed different performance were lexical decision tasks and incidental recall tasks. These are not perceptual tasks by any means. The speeding up of response time when thirsty may have been due to any post-perceptual stages of the processing that goes into these complex and cognitively demanding tasks. In other words, while Aarts and colleagues talk about perceptual readiness, there is little evidence that there is anything perceptual about this (and don’t get me started on ‘readiness’…)

The second experiment one might expect to be mentioned in this context is by Gallistel (1980). Gallistel examined patients whose eye muscles are paralysed. His most interesting finding from our point of view is the following:

When a man with paralyzed eye muscles tries to glance to the right the world appears to jump to the right even though the pattern of light falling on the paralyzed eye has not moved. […] The image of the world on the retina does not move, but one “sees” the world move. Gallistel 1980, p. 175.^4^

When a patient tries to move her eyes, an action she attempts to perform intentionally and voluntarily, her sensory stimulation does not change, but her perceptual experience does—it appears to jump to the right. In other words, her perceptual experience varies as her intention varies, even if the sensory stimulation stays the same.

This is an indicative finding, but I will not rely on it for my argument because there is no evidence that it is the intention to act that influenced perceptual phenomenology and not the action itself. Also, given that the action in question is a mental action, there are tricky questions to answer about the relation between the intention to act and acting per se.

The most important and most directly relevant empirical finding about the influence of intention on perception is from Tjerk Gutteling et al. (2015). In their experiment they varied the action the subject intended to perform (grasping versus pointing) and this action preparation influenced the early visual cortices (and even the primary visual cortex). It is important that the actions were not in fact executed: action preparation (what philosophers would call ‘proximal intention’) influenced early visual processing. Importantly, subjects’ retinal image remained the same as they were fixating on the same point in both tasks.

This is a very important finding, but from a philosophical point of view one would want to know how this influence happens and what mediates between intentions and perception. And my answer is that this mediator is attention. So we get the following picture:
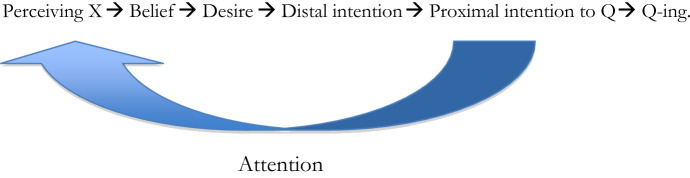


## The first step: attention and intention

What I need to show is that one’s perceptual processing depends counterfactually on one’s intention to perform an action (everything else being equal). I will show this in two steps:

(i) one’s perceptual attention depends counterfactually on one’s intention to perform an action (everything else being equal) and
(ii) one’s perceptual processing depends counterfactually on one’s perceptual attention (everything else being equal).

The conclusion then would be (bracketing worries about transitivity I will return to in Sect. 6) that:

(iii) one’s perceptual processing depends counterfactually on one’s intention to perform an action (everything else being equal)

I take the first of these two steps to be relatively uncontroversial: what we attend to obviously depends on what action we intend to perform. I will attend to different features of my phone when I intend to call a cab with it and when I intend to throw it out of the window. There is one possible worry about this first step though: that what we attend to depends on what action we intend to perform *even if the sensory stimulation is the same*. One could argue that I attend to different features of my phone when I intend to call a cab with it and when I intend to throw it out of the window *because* my sensory stimulation is different. After all, our eye movements are going to be very different in the two situations. And one may argue that if the eye movements are different, then the sensory stimulation is also different.

The worry is about the relation between attention and eye movements. One might think that difference in attention implies difference in eye movement—in other words, that attention supervenes on eye movement. But then we run into a problem. If attention supervenes on eye movement, it cannot possibly vary with intention while the eye movement (thus, the sensory stimulation) is the same. Thus, (i) is false.

The assumption that attention supervenes on eye movement, however, is incorrect. Change in attention is possible without any change in eye movement. So much so that this very phenomenon has been widely investigated under the name of ‘covert shift of attention’ (Posner 1980, 1984; Posner et al. 1984; see also Findlay and Gilchrist 2003).

It has even been argued that eye movement would not even be possible without this covert shift of attention. When the eyes move, they must be preceded by a covert shift of attention, because, to put it very simply, otherwise our visual system would not know whether and how it should move the eyes (see, for example, Hoffman and Subramaniam 1995; Kowler et al. 1995).

The existence of the phenomenon of covert shift of attention is universally agreed on (see Findlay and Gilchrist 2003, pp. 35–54 for a good summary). It is possible to shift one’s attention while not moving one’s eye (that is, while having the same sensory stimulus). Thus, attention does not supervene on eye movement. It is possible that our attention varies while our eye movement is the same. Thus, it is also possible that it varies as our intention changes (while our eye movement is the same). So much about the first step of my argument.

## The second step: perception and attention

The second step of my argument is, however, more problematic. Again, what I need to show is that one’s perceptual processing depends counterfactually on one’s perceptual attention (everything else being equal). This suggestion is not new. William James, for example, says: “In a world of objects thus individualized by our mind’s selective industry, what is called our ‘experience,’ is almost entirely determined by our habits of attention.” (James 1892/1961, p 39, see also Nanay 2010).

Again, one may think here of the widely popularized inattentional blindness literature that shows how much perception is influenced by attention (Simmons and Chabris 1999; Mack and Rock 1998; Koivisto et al. 2004; but see also Nanay 2016 for a philosophical summary). One of the most celebrated inattentional blindness experiments is the following. You are shown a short clip of people playing basketball: a team dressed in white against a team dressed in black. Your task is to count how many times one of the teams passes the ball around. While doing this, more than half of the people fail to notice that a man in a gorilla costume walks in the frame, makes funny gestures, spends seven full seconds there and then leaves. If you’re not trying to do any counting tasks, you immediately spot the gorilla. So what you’re attending to has serious consequences for whether you spot a man in a gorilla costume bang in the middle of the screen.

The inattentional blindness experiments are about perceptual experience—a concept I am trying to stay safely away from. And it is exactly the concept of perceptual experience that casts some doubts on how useful these experiments are for establishing the claim that perceptual attention influences perception. It has been suggested that attention does not influence perception. The subjects perceive the gorilla all right. But they forget it immediately (Wolfe 1999). As it is difficult to argue about perceptual phenomenology, this debate, as long as it is understood to be about perceptual experience, is difficult to resolve. (We have some evidence that inattentional blindness influences not just visual experience, but also visual processing (see, for example, Rees et al. 1999), but I leave this possible way of arguing for my conclusion aside).

More generally, there is full agreement in neuroscience that attention modulates and influences processing already in the primary visual cortex (Watanabe et al. 2011; Murray et al. 2002; Gandhi et al. 1999; Kok et al. 2012, 2014) and even the thalamus (O’Connor et al. 2002). These results are much less difficult to quibble with than the inattentional blindness experiments (Summerfield and De Lange 2014; Summerfield and Egner 2009; Teufel and Nanay 2017 for summaries). In short, perceptual attention influences early cortical perceptual processing.

In the cognitive penetration debate, attention is a hotly debated topic. Those who argue that perception is not cognitively penetrable would allow for attentional influences on perception (see Pylyshyn 1999) but argue that these attentional effects are consistent with the impenetrability of perception as the influence precedes the modularist processing. The general idea is that attention changes the input. So it is not the case that beliefs influence perception while the sensory stimulation remains the same as the sensory stimulation does not remain the same as a result of changes in attention.

It has been pointed out that this argument presupposes that attention is always overt attention and as we know (see last section), it’s not (Mole 2015; see also Gross 2017; Stokes 2018). It can be covert: we can shift our attention without moving our eyes. If so, then attention can be the mediator of cognitive penetration. And, as I argued, it can also be the mediator in the influence of intentions on perception. But in order to conclude this argument, I need to say more about how the first and the second premise of the argument fits together.

## Some final worries about transitivity

Perceptual processing depends counterfactually on one’s perceptual attention (even if the sensory stimulation is the same). Further, one’s perceptual attention depends counterfactually on the action one intends to perform (even if the sensory stimulation is the same). If we put these two claims together, what we get is that perceptual processing depends counterfactually on the action one intends to perform (even if the sensory stimulation is the same).

A possible worry is raised by the transitivity of counterfactual dependence. The structure of my argument is that A depends counterfactually on B and B depends counterfactually on C, therefore, A depends counterfactually on C. David Lewis, however, famously argued that counterfactual dependence is not always transitive (Lewis 1973, pp. 32–35). If P depends counterfactually on Q and Q depends counterfactually on R, then it is possible that P does not depend counterfactually on R if what we hold fixed in the first counterfactual is different from what we hold fixed in the second. For example, I would not have ducked if the boulder had not come careering down the mountain slope. I would not have survived if I had not ducked. But it is not the case that I would not have survived if the boulder had not come careering down the mountain slope.

Thus, there are cases where we are not entitled to make the inference that if P depends counterfactually on Q and Q depends counterfactually on R then P depends counterfactually on R. I need to show that in my argument I am indeed entitled to make such inference. The reason why the counterfactuals in the boulder example are not transitive is that what we hold fixed in the first counterfactual is not the same as what we hold fixed in the second. The second counterfactual can be rephrased in the following way: I would not have survived if I had not ducked, other things (most importantly, the careering boulder) being equal. Part of what we hold fixed in this counterfactual is that boulder comes careering down towards me. This is obviously not something we hold fixed in the first counterfactual, since this very fact is what my ducking depends on counterfactually.

In my argument, there are no such complications. What I hold fixed in the two counterfactuals, most importantly, that the sensory stimulation does not change, is the same. If it is true that perceptual attention depends on proximal intentions counterfactually and if it is also true that perceptual processing depends on perceptual attention counterfactually, we can conclude, given transitivity, that perceptual processing depends on proximal intentions counterfactually.

## Conclusion

Perception depends on attention; attention depends on intention. So perception depends on intention. If we grant that perceptual processing depends on intention, there is a further question to ask: Does the action we have the intention to perform show up somehow in the content of our perceptual state? There may be reasons to think that it does, at least sometimes (Nanay 2011a, b, 2012a, b and also Jeannerod 1997; Clark 1995; Siegel 2015 for some less self-indulgent references). But nothing I said in this paper presupposes that this is so. My claim was more modest: perceptual processing is influenced by intention, regardless of whether perceptual content is.

The conclusion is that perception depends on intention. This dependence bypasses our beliefs (in the sense that intention does not merely influence perception by influencing beliefs and then the beliefs influence perception). Regardless of what we think of the cognitive penetrability debate, we have strong reasons to hold that perception is not all-purpose.

## Footnotes

1 But see also Wu (2013) on the way perception and intention interact in the context of perceptual constancies.

2 In fact, very different. More particularly, Searle’s distinction may not map onto the others mentioned above as he takes intention-in-action to be part of the action, whereas proximal intention, immediate intention and present-directed intention trigger actions, but are not themselves part of the action (see Mele 1992, p. 181, see also O'Shaughnessy 1991; Pacherie 2001 for good overviews on the concept of intention in action).

3 As Mele writes, “the acquisition of a proximal intention […] initiates the sending of a ‘command’ signal. Mele sometimes (especially in Mele 1992) writes about proximal intentions as *propensities* to execute an action (and a propensity to do Q does not imply doing Q). An example: “A proximal intention to do an A incorporates a propensity of the agent to execute a certain representational component of the [distal] intention (Mele 1992, p. 176). But I take it that Mele’s official account is that proximal intentions in fact trigger the action—thus the often-repeated phrase of “the acquisition of proximal intention triggers appropriate actional mechanisms (see, for example, Mele 1992, p. 181, footnote 2).

4 See also Howard (1982, p. 311) and Hurley (1998, pp. 372–373) on the paralyzed eye experiments.
